# Variant Selection and Interpretation: An Example of Modified VarSome Classifier of ACMG Guidelines in the Diagnostic Setting

**DOI:** 10.3390/genes12121885

**Published:** 2021-11-25

**Authors:** Francesca Cristofoli, Elisa Sorrentino, Giulia Guerri, Roberta Miotto, Roberta Romanelli, Alessandra Zulian, Stefano Cecchin, Stefano Paolacci, Jan Miertus, Matteo Bertelli, Paolo Enrico Maltese, Pietro Chiurazzi, Liborio Stuppia, Marco Castori, Giuseppe Marceddu

**Affiliations:** 1 Diagnostics Unit, MAGI EUREGIO, 39100 Bolzano, Italy; francesca.cristofoli@assomagi.org (F.C.); elisa.sorrentino@assomagi.org (E.S.); roberta.miotto@assomagi.org (R.M.); jan.miertus@assomagi.org (J.M.); matteo.bertelli@assomagi.org (M.B.); giuseppe.marceddu@assomagi.org (G.M.); 2 Diagnostics Unit, MAGI’S LAB, 38068 Rovereto, Italy; giulia.guerri@assomagi.org (G.G.); roberta.romanelli@assomagi.org (R.R.); alessandra.zulian@assomagi.org (A.Z.); laboratorio@assomagi.org (S.C.); paolo.maltese@assomagi.org (P.E.M.); 3 Section of Genomic Medicine, Department of Life Science and Public Health, “Sacro Cuore” Catholic University, 00168 Rome, Italy; pietro.chiurazzi@unicatt.it; 4 Policlinic University Foundation “A. Gemelli” IRCCS, UOC Medical Genetics, 00168 Rome, Italy; 5 Department of Psychological, Health and Territorial Sciences, School of Medicine and Health Sciences, “G. D’Annunzio” University, Chieti-Pescara, 66100 Chieti, Italy; stuppia@unich.it; 6 Division of Medical Genetics, IRCCS Foundation “Casa Sollievo della Sofferenza”, 71013 San Giovanni Rotondo, Italy; m.castori@operapadrepio.it

**Keywords:** variant interpretation, ACMG, VarSome

## Abstract

Variant interpretation is challenging as it involves combining different levels of evidence in order to evaluate the role of a specific variant in the context of a patient’s disease. Many in-depth refinements followed the original 2015 American College of Medical Genetics (ACMG) guidelines to overcome subjective interpretation of criteria and classification inconsistencies. Here, we developed an ACMG-based classifier that retrieves information for variant interpretation from the VarSome Stable-API environment and allows molecular geneticists involved in clinical reporting to introduce the necessary changes to criterion strength and to add or exclude criteria assigned automatically, ultimately leading to the final variant classification. We also developed a modified ACMG checklist to assist molecular geneticists in adjusting criterion strength and in adding literature-retrieved or patient-specific information, when available. The proposed classifier is an example of integration of automation and human expertise in variant curation, while maintaining the laboratory analytical workflow and the established bioinformatics pipeline.

## 1. Introduction

High-throughput DNA sequencing technologies have allowed diagnostic laboratories to offer unprecedented molecular testing for a wide range of rare diseases at lower costs and with faster and more conclusive results than the variant-specific approach or even a gene-by-gene Sanger sequencing strategy. In the diagnostic setting, massive parallel sequencing of targeted multigene panels, exomes, or even entire genomes uncovers a vast number of rare and often private variants per individual. These should be always interpreted in relation to the clinical context in which the necessity of the molecular test arose. Therefore, the challenge for diagnostic laboratories is moving from quality issues related to technical standards to assessing the impact of identified variants for the presenting phenotype and, ultimately, making this information available for medical purposes, including diagnosis, prognostication, and, as expected in an increasing number of conditions, treatment.

To address this challenge, in 2008 and, again, 2015, the American College of Medical Genetics and Genomics (ACMG) and the Association for Molecular Pathology (AMP) revised and updated its original guidelines [1,2] for the interpretation of sequence variants identified through genomic testing in patients with suspected inherited Mendelian disorders [3]. These guidelines recommend the use of a five-tier system of classification for sequence variants (pathogenic, likely pathogenic, uncertain significance (VUS), likely benign, and benign) and provide a set of 28 criteria that organize the available levels of information (i.e., population data, computational and predictive data, functional data, segregation data, and other). Sixteen rules evaluate evidence towards a pathogenic interpretation, and another 12 towards a benign classification. The criteria are in turn weighted according to the strength of the available evidence: very strong, strong, moderate, and supporting for pathogenicity; stand-alone, strong, and supporting for benignity. Eventually, their combination enables the final clinical interpretation of any variant. In this context, reproducibility and consistency of interpretation of identified variants are crucial for translational issues, while the increasing burden related to VUS reporting adds complexity to rather than supporting medical decisions.

The limits of the ACMG/AMP guidelines in being applied in all laboratories and all clinical scenarios were evident since their publication [4,5]. Terms such as “mutational hotspot” or “well-established” as defined in the 2015 paper are intrinsically too broad and clearly need to be specified case by case. Accordingly, some diagnostic laboratories developed tailored adaptations of the original classification system for the attribution or exclusion of criteria related to public databases information. For instance, Nykamp et al. [6] developed Sherloc, a method that allows a robust evaluation of evidence supported by a set of hierarchical decision trees to provide a more transparent and reproducible approach to variant classification. A second example is the rule-based iterative scoring method developed by Karbassi et al. [7] that provides a variant pathogenicity risk score based on clinical grade information and offers a more stable variant interpretation system.

The issue of subjectivity of the ACMG/AMP guidelines also extends to the application and strength attribution of criteria related to the clinical setting (e.g., specificity of the phenotype—PP4, co-segregation with the phenotype, PP1, de novo origin, PM6/PS2). For these reasons, a quantitative approach has been subsequently proposed for the consideration of the co-segregation criterion [8]. The 2015 guidelines have been also converted to a Bayesian framework that provides a mathematical foundation to the inevitably general recommendations, thus supporting efforts to automate certain components of variant pathogenicity assessment [9]. Furthermore, the ClinGen Sequence Variant Interpretation (SVI) Working Group has developed detailed specifications for criteria such as PVS1 [10], BA1 [11], PP5/BP6 [12], and PS3/BS3 [13]. Many Variant Curation Expert Panel (VCEP) working groups have elaborated ad hoc interpretation guidelines for specific disorders [14,15], single genes [16,17], and mitochondrial [18] or other well-characterized variants [19].

Finally, as public database consultation remains a critical step in formulating the final interpretation before reporting, the consistency of published information is crucial for the clinical application of stored data. Accordingly, curation efforts have been made to understand and reconcile discrepancies between variants deposited in common repositories such as the ClinVar database. These studies have demonstrated the importance of data sharing to facilitate the identification of differences and to expertly reassess pathogenicity, thus improving testing quality and patient care [20,21].

In 2016, with the advent of VarSome [22], a suite of bioinformatic tools for processing and annotation of NGS data, aggregated information from multiple databases and sources became available to the global genomics community, helping users to assess variants in their genomic context. In a previous publication, we described the integration of the VarSome Application Programming Interface (API) into the NGS data analysis workflow of our molecular genetics’ laboratory [23]. Here, we describe our adapted application of the VarSome variant classifier to the reporting workflow and discuss its strengths and pitfalls in the field of rare Mendelian disorders. While acknowledging the strengths of the VarSome classifier, the need to incorporate the automated classifier into the laboratory pipeline [24] to guarantee sample traceability and high-quality standards in the diagnostic setting prompted us to develop an internal ACMG-based variant classifier that retrieves VarSome automated criteria and performs variant interpretation according to the recommended ACMG combinatory rules, with some modifications. After review by a molecular geneticist who can also perform any necessary criterion or strength adjustment according to an ACMG-based criterion checklist, the algorithm recalculates the final variant interpretation.

## 2. Materials and Methods

### Filtering Benign/Likely Benign Variants through VarSome Stable-API

VarSome API is a high-performance variant annotation tool that can be queried to extract information aggregated from a wide variety of databases. We used the Stable-API environment to retrieve information on variants identified through panel-based NGS in affected individuals (singleton analysis) referred to our laboratory for molecular testing. The Stable-API environment was selected because it allows greater interpretative stability while maintaining the bioinformatics pipeline set up of our laboratory [25]. Stable-API documentation is available at the following link: https://stable-api.varsome.com/ (accessed on 15 April 2021).

Variants with a Minor Allele Frequency (MAF) below 3% are selected for annotation through VarSome Stable-API. The 3% threshold was chosen as a good compromise between 1%, which defines the term “polymorphism” versus “mutation”, and the computational burden of analyzing hundreds of variants, most of which are neutral. The “DecisionMAF” is calculated by integrating data retrieved from dbNSFP [26], VEP [27], and gnomAD [28]. In general, “DecisionMAF” is the highest variant frequency among available subpopulations, excluding the Finnish, Ashkenazi, and other groups. The baseline setting of the VarSome classifier was considered consistent for the interpretation of “benign” and “likely benign”. Therefore, all variants with an automatic “benign or likely benign” verdict are not considered for further analysis, except for the set of variants recommended by Ghosh et al. [11], and two known hypomorphic variants in *ABCA4* and *TYR*. Variants with uncertain, likely pathogenic, and pathogenic automatic verdict are kept with the criteria assigned by VarSome and sent to the internal ACMG-based classifier [23]; modifications are described in Table 1 and Results. After manual review and adjustment of unimplemented criteria (BS3, BS4, BP2, BP5, PS2, PS3, PS4, PM3, PM6, PP1, PP4) by the molecular geneticist according to the specifications of Table 2, the final variant interpretation is obtained.

The code for this project is available at https://gitlab.com/magieuregio/automate_variant_interpretation (accessed on 15 April 2021). All variants discussed in the paper were interpreted using VarSome Stable-API v.9.4.6.

## 3. Results

Table 2 shows the internal ACMG-based variant classification checklist with the indications to be followed by the molecular geneticist to modify the criteria assigned automatically in the VarSome Stable-API environment and to assign the unimplemented criteria. We then describe some features of the checklist in greater depth.

### 3.1. Varsome Algorithm Modifications

In VarSome, any Benign Strong (BS) rule is sufficient to interpret a variant as likely benign. In our algorithm, if a single BS criterion (BS1–BS4) is triggered together with a pathogenic criterion (at any strength), the former is not considered in the combinatory rules to reach the final verdict. Moreover, since only three of the five Pathogenic Supporting (PP) rules can be automated, one Pathogenic Moderate (PM) and three PP rules are sufficient to trigger a likely pathogenic verdict, while for ACMG recommendations, one PM and four PP are required to obtain the same verdict. We kept these modifications in our internal algorithm to avoid differences in variant selection (see Table 1). However, we did not allow any Benign criterion to be set to “Moderate” or “Very Strong”, as these strengths are not foreseen in the ACMG guidelines. Therefore, if a Benign criterion was assigned a Moderate or Very Strong level, we brought its strength back to its standard value (i.e., Supporting or Strong). Usually, only the BP6 criterion is found significantly boosted to Moderate or Very Strong in the VarSome algorithm if evidence reported in ClinVar or by VarSome users justifies it.

According to the ACMG/AMP guidelines, criteria listed as one weight can be moved to another weight using professional judgment. We therefore instructed the internal classifier that also two Very Strong criteria lead to a pathogenic verdict and to include the possibility that other pathogenic criteria might be upgraded to Very Strong if evidence justifies it. We also instructed the classifier that a Pathogenic verdict is reached with the combination 1PVS and 1PM and one or multiple PP criteria, and that PS and PM criteria can also be downgraded to Supporting.

### 3.2. Exceptions to BA1: Hypomorphic Variants

In our laboratory, a large group of patients referred for clinical testing present with eye disorders, such as retinal dystrophies. A significant proportion of these patients, especially those suspected to have Stargardt disease/cone–rod dystrophy, bear pathogenic variants in the *ABCA4* gene. Currently, more than 1200 disease-causing variants of this gene are known, varying in type (missense, nonsense, canonical splicing, deep intronic) and severity (from fully penetrant to hypomorphic) [30]. The most prominent hypomorphic variant is c.5603A>T p.(Asn1868Ile) [rs1801466] which has a frequency of about 7% in the European general population. This variant would therefore be assigned the BA1 criterion (frequency > 5%) and classified as benign; however, its pathogenicity was proved by Zernant et al. [31] when it is in trans with a fully penetrant variant, such as a nonsense mutation predicted to undergo NMD.

The second hypomorphic variant that we report is c.1205G>A p.(Arg402Gln) in the *TYR* gene (rs1126809). This variant has a MAF of about 27% in the gnomAD European Non-Finnish population, which would be sufficient to assign the BA1 criterion and classify it as benign. However, functional studies have demonstrated that this is a thermosensitive variant with reduced activity at normal body temperature. This variant is associated with mild forms of oculocutaneous albinism in some compound heterozygous individuals who bear a second pathogenic variant, suggesting that it causes partial albinism only when paired with certain genetic backgrounds [32,33]. We report both these variants as phenotype modifiers.

### 3.3. In-Depth Evaluation of the PVS1 Criterion

The strongest pathogenicity criterion (PVS1) in the ACMG guidelines may be attributed to a “null variant in a gene where LoF is a known mechanism of disease”. There were few original recommendations, such as the warning not to apply the criterion for genes where LoF is not a known disease mechanism, using caution for variants affecting the extreme 3′ end of a gene, or for splice variants that are predicted to lead to exon skipping but leave the remainder of the protein intact. Since these recommendations were quite limited, a subsequent publication offered more detailed guidelines and provided a complete decision-making scheme for applying the PVS1 criterion. The proposed decisional pathways take into consideration information about variant type, location, disease mechanism, and other additional evidence to determine the likelihood of a true null effect [10].

The complexity of PVS1 specifications makes it challenging to aggregate the information required computationally and tune criterion strength. In our laboratory, this evaluation was therefore done manually. Recently, however, an online tool was developed to automatically streamline the PVS1 decisional pathway and support molecular geneticists in a preliminary evaluation of the best strength level for PVS1 [34]. This tool can be used to identify the most appropriate PVS1 strength level for LoF variants.

**Figure 1:**
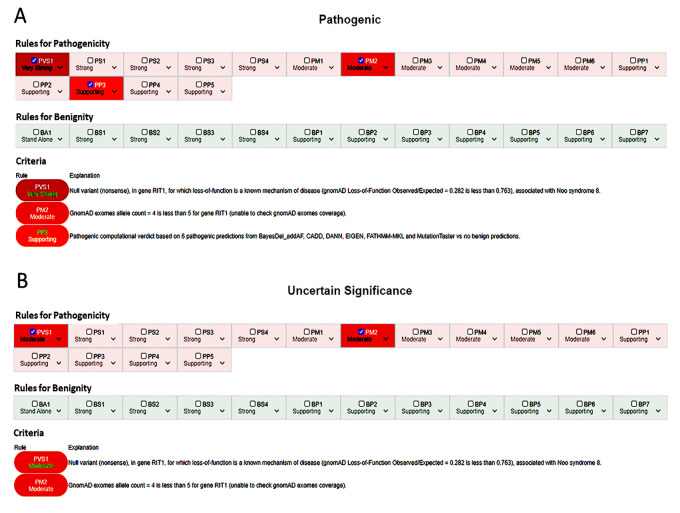
(**A**) Automatic classification for the c.650C>G p.(Ser217*) variant in the *RIT1* gene using VarSome Stable-API v.9.4.6. (**B**) Adjusted manual interpretation with PVS1_Moderate and PP3 disabled according to ACGS 2019 recommendations [29].

**Figure 2:**
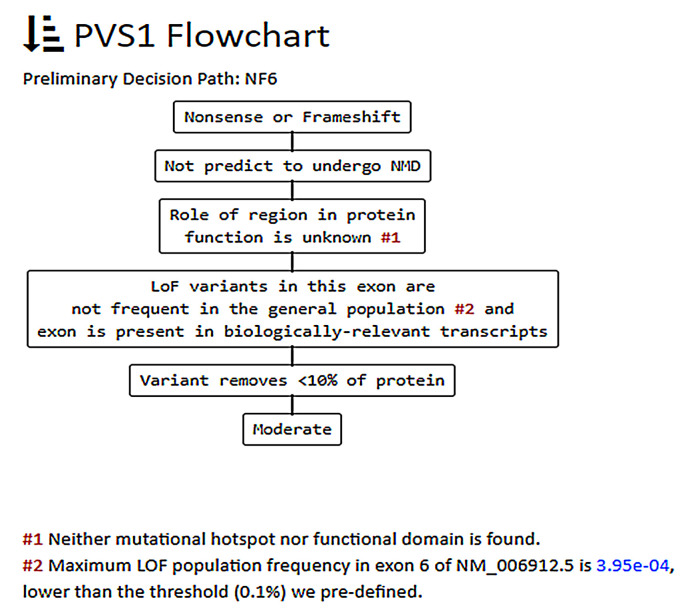
Preliminary decision path for the c.650C>G p.(Ser217*) variant in the *RIT1* gene, as calculated by the AutoPVS1 tool [34].

For instance the c.650C>G p.(Ser217*) variant in the *RIT1* gene, associated with Noonan syndrome 8 (MIM #615355), may be classified as pathogenic if PVS1 is used at a very strong level (Figure 1A); however, more detailed evaluation according to the guidelines provided by Abou Tayoun et al. [10] through the AutoPVS1 algorithm suggests using the criterion at moderate level (see Figure 2), thus leading to an uncertain significance interpretation (Figure 1B).

### 3.4. PM1 Criterion (Mutational Hotspot and/or Critical and Well-Established Functional Domain, without Benign Variation)

**Figure 3:**
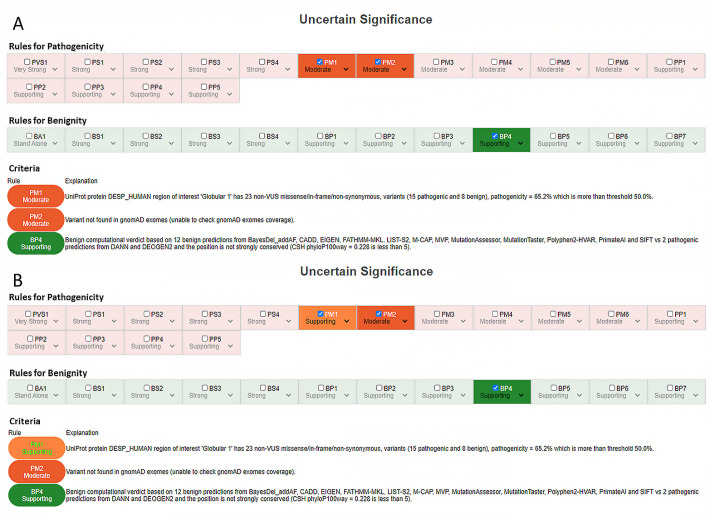
(**A**) Automatic classification for c.2582C>T p.(Thr861Ile) in the *DSP* gene and (**B**) downgrading of PM1 to Supporting due to the length of the “Globular 1“ region of interest (1–1056 AA).

A mutational hotspot is defined by VarSome as a region of 25 base pairs on either side of the variant of interest. The rule is assigned if there are at least six pathogenic missense or in-frame indel variants; their distance is also weighted by computing a “proximity score”. The functional domains considered are those reported by UniProt. The rule considers clinically reported variants within the domain and is assigned if the ratio of pathogenic to total non-VUS variants is greater than 0.5, with at least one pathogenic variant within the domain. We established that this criterion can be downgraded to Supporting, especially when the pathogenic variants reported in the functional domain are less than 10 or when these variants are scattered in a very large UniProt domain. For example, the variant c.2582C>T p.(Thr861Ile) in the DSP gene (NM_004415) is interpreted as VUS for criteria PM1, PM2, and BP4 (VarSome Stable-API v.9.4.6.), and the PM1 criterion is assigned considering the 23 non-VUS missense/in-frame/non-synonymous variants (15 pathogenic and 8 benign) in the UniProt region of interest “Globular 1”, which is 1056-amino acid long. This would lead the geneticist to downgrade the criterion to Supporting or even exclude it, according to the internal checklist (Table 2), without changing the variant’s final interpretation (Figure 3). This might not seem important in the light of the ACMG five-tier classification system; however, it may become relevant for the finer differentiation of VUS (from ice-cold to hot) proposed by the ACGS 2020 guidelines [35,36].

Attention must be also paid to variants that have been automatically assigned the PM1 criterion because they are located in a “disulfide bond domain”. In this case, we would not activate PM1, as the disulfide bond only involves the two cysteine residues forming the bridge. For instance, the c.151G>T p.(Asp51Tyr) variant in the *CTSC* gene (NM_001814) or the c.514G>A p.(Gly172Ser) variant in the *ABCA4* gene (NM_000350) are assigned PM1 because the former is located in “disulfide bond_30–118”, and the latter in “disulfide bond_75–324”, connections that only affect the residues at their edges. By contrast, for the in-frame variant c.1024_1032del p.(Glu342_Asn344del) in the *CRB1* gene, PM1 is assigned because the variant is located in the “EGF-like 9” domain (AA 339–395) with four non-VUS missense/in-frame/non-synonymous pathogenic variants (versus one benign). Although these values would allow us to downgrade the criterion to Supporting, more detailed evaluation shows that the deleted residue Cys343 is involved in a disulfide bond with Cys354, leading to disruption of a disulfide bridge that might be important for the protein’s structure and function.

### 3.5. PP3/BP4 Criteria (Functional Predictors)

For nonsense and frameshift variants where PVS1 can be assigned, we do not activate the PP3 or PM4 criteria, as specified in the ACGS Best Practice Guidelines for Variant Classification 2019 [29]. PP3 can be assigned to intronic variants outside the canonical splice sites if splicing predictors ADA and RF scores are above the following cutoffs: ADA score > 0.708; RF score > 0.515. We also turn off PP3/BP4 if these criteria were assigned on the basis of a single predictor, as we established that at least three total and concordant predictors are required to assign them.

### 3.6. Observations on Genes and Associated Inheritance Patterns

Clinically relevant information in VarSome is retrieved from the NIH Clinical Genomic Database, a manually curated database of conditions with known genetic causes (https://research.nhgri.nih.gov/CGD/, accessed on 15 April 2021). The inheritance patterns of disorders associated with a specific gene are then used to discern whether the gene should be classified as “dominant” or “recessive”: in the first case—or if both AD/AR inheritance patterns have been observed for the gene and for X-linked disorders—the PM2 rule (i.e., absent from controls or at extremely low frequency if recessive) is triggered if the gnomAD allele count is less than five. For AR inheritance only, PM2 is triggered if the number of homozygous individuals in the gnomAD database is fewer than three. The same thresholds are used to assign the BS2 criterion [i.e., “Observed in a healthy adult for a recessive (homozygous), dominant (heterozygous), or X-linked (hemizygous) disorder, with full penetrance expected at an early age)]. Therefore, caution is needed when evaluating variants in genes associated with AD and AR disorders, especially if the suspected diagnoses are different, as the underlying pathogenic mechanisms might be different or the disorders may be caused by different types of mutations (e.g., LoF versus dominant negative) or by missense variants affecting specific domains of the protein.

For instance, the *PIEZO1* gene (NM_001142864) is associated in the OMIM database with Dehydrated hereditary stomatocytosis with or without pseudohyperkalemia and/or perinatal edema (OMIM #194380), an AD hemolytic anemia characterized by primary erythrocyte dehydration. However, the gene has also been associated with Lymphatic malformation-6 (OMIM #616843), a form of generalized lymphatic dysplasia that affects all segments of the body, with systemic involvement such as intestinal and/or pulmonary lymphangiectasia. This lymphatic disorder has AR inheritance. Currently, the inheritance pattern retrieved from CGD for the *PIEZO1* gene is dominant; therefore, when filtering variants for suspected lymphatic malformation one might miss variants with more than five alleles but less than three homozygotes, as PM2 would not apply, while BS2 would be assigned instead. In our laboratory, we have seen a patient with primary lymphedema bearing the two missense variants c.1447G>A p.(Val483Met) and c.5891T>C p.(Met1964Thr) in the *PIEZO1* gene. The first variant is automatically interpreted as likely benign for BS2 based on the allele count in gnomAD genomes v.3.1.1 (*n* = 13), while the second is automatically interpreted as of uncertain significance (VUS) for PM2 (one allele in gnomAD genomes v.3.1.1) and PP3 (seven pathogenic versus five benign predictors). However, we would interpret the c.1447G>A p.(Val483Met) variant as VUS considering a recessive inheritance pattern and assign the PM2 criterion instead, thus including it in the patient’s report. Segregation analysis in family members might improve the interpretation of these variants in this subject.

### 3.7. Late-Onset Disorders

For late-onset disorders, detection of a variant in a healthy adult should not be considered strong evidence of a benign variant, as the “healthy” subject bearing it might not yet have manifested the disorder. For instance, the onset of AD vitelliform macular dystrophy (OMIM #153700) associated with mutations in the *BEST1* gene may vary from infancy to adulthood, while AD vitreoretinochoroidopathy (OMIM #193220) and AR bestrophinopathy (OMIM #611809) usually manifest in the first decade of life [37]. Therefore, VarSome disables rule BS2 for the *BEST1* gene, and this makes it possible to retrieve variants with more than five alleles in the population database, as in the CGD database only dominant inheritance is highlighted, with bestrophinopathy mentioned as an allelic condition. When family history and clinical findings are consistent with AR bestrophinopathy and there are additional pathogenic criteria supporting that the variant might not be clinically neutral, *BEST1* variants should be assigned PM2 according to the established AR cutoff (i.e., three homozygotes).

A second example concerns the *CTNNA1* gene. Loss-of-function (LoF) mutations are associated with hereditary diffuse gastric cancer syndrome [38,39], while missense mutations cause butterfly-shaped pigmentary macular dystrophy type 2 (OMIM #608970), an AD adult-onset eye disease characterized by macular lesions that can resemble the wings of a butterfly. The disorder is generally benign but can progress to a more severe phenotype and vision loss [40,41]. In the CGD database, this disorder is considered an allelic condition of the oncological phenotype. Despite this, missense variants in *CTNNA1* for which the PM2 criterion is not met (i.e., at least or more than five alleles in the gnomAD database) do not escape selection because BS2 is not triggered, since the oncologic phenotype is an adult-onset disorder.

## 4. Discussion

We have described the development of an internal ACMG-based variant interpretation system used in our laboratory to assist molecular geneticists in refining variant classification. First, a pre-selection of annotated variants is made using VarSome Stable-API. Only uncertain, likely pathogenic, and pathogenic variants are then kept and submitted to an internally developed ACMG-based classifier that uses the criteria automatically assigned through VarSome Stable-API to re-interpret the selected variants. This system allows the geneticist to introduce the necessary modifications to criteria strength, add other unimplemented criteria when available, and obtain the final variant classification. We drew up an ACMG-based checklist that contains indications on how to vary criteria strength and how to add functional or co-segregation information. We applied this variant interpretation system in the diagnostic pipeline of our laboratory. It allowed us to maintain the analytical workflow that we developed over the years to ensure sample traceability and quality management from sample collection to clinical report generation [25]. The combination of data retrieved from VarSome and detailed curation by the molecular geneticist according to the ACMG-based checklist enables a high-quality variant classification through integration of automation and human expertise.

## 5. Conclusions

We emphasize the importance and feasibility of combining information that can be provided by VarSome, through its extensive aggregation of databases and sources, with human expertise in the classification of genetic variants. To our knowledge, this work represents the first attempt to integrate the widely employed VarSome variant annotation and interpretation tool into a pre-existing diagnostic workflow, adding further specifications to the ACMG criteria to internally standardize variant interpretation.

## Figures and Tables

**Table 1:** Combination of criteria for variant interpretation according to ACMG-AMP guidelines. The modifications implemented in the internal ACMG-based classifier are underlined.

Pathogenic	(i) 1 Very strong (PVS1) AND (a) ≥1 Strong (PS1–PS4) OR (b) ≥2 Moderate (PM1–PM6) OR (c) 1 Moderate (PM1–PM6) AND at least * 1 Supporting (PP1–PP5) OR (d) ≥2 Supporting (PP1–PP5) OR (e)≥1 Very strong (PVS1) * (ii) ≥2 Strong (PS1–PS4) OR (iii) 1 Strong (PS1–PS4) AND (a)≥3 Moderate (PM1–PM6) OR (b)2 Moderate (PM1–PM6) AND ≥ 2 Supporting (PP1–PP5) OR (c)1 Moderate (PM1–PM6) AND ≥ 4 supporting (PP1–PP5)
Likely Pathogenic	(i) 1 Very strong (PVS1) AND 1 moderate (PM1– PM6) OR (ii) 1 Strong (PS1–PS4) AND 1–2 moderate (PM1–PM6) OR (iii) 1 Strong (PS1–PS4) AND ≥ 2 supporting (PP1–PP5) OR (iv) ≥3 Moderate (PM1–PM6) OR (v) 2 Moderate (PM1–PM6) AND ≥ 2 supporting (PP1–PP5) OR (vi) 1 Moderate (PM1–PM6) AND ≥ 3 supporting (PP1–PP5)
Benign	(i) 1 Stand-alone (BA1) OR (ii) ≥2 Strong (BS1–BS4)
Likely benign	(i) 1 Strong (BS1–BS4) ° OR (ii) ≥2 Supporting (BP1–BP7)
Uncertain significance	(i) Other criteria shown above are not met OR (ii) the criteria for benign and pathogenic are contradictory

* These modifications were added to include the possibility of varying the strength of pathogenic criteria. ° If ONLY a strong benign criterion (BS1–BS4) is triggered together with any pathogenic criterion, the former is not considered in deciding the final verdict.

**Table 2:** Combination of criteria for variant interpretation according to ACMG-AMP guidelines and modifications implemented in the internal ACMG-based classifier.

BENIGN CRITERIA		
ACMG CRITERIA	ASSIGNED BY VARSOME	EXCEPTIONS AND INDICATIONS
BA1 Allele frequency > 5% in Exome Sequencing Project, 1000 Genomes Project or Exome Aggregation Consortium	YES	Variants recommended in Ghosh et al. 2018 - Hypomorphic variants: NM_000350 (*ABCA4*):c.5603A>T (p.Asn1868Ile) NM_000372 (*TYR*):c.1205G>A (p.Arg402Gln)
BS1 Allele frequency greater than expected for disorder	YES (not evaluated if BA1 or PM2 are activated)	Use STRONG as default. For AD diseases with high penetrance, the criterion can be used as STAND-ALONE evidence (sufficient to classify a variant as likely benign).
BS2 Observed in a healthy adult for a recessive (homozygous), dominant (heterozygous), or X-linked (hemizygous) disorder, with full penetrance expected at an early age	YES (not evaluated if BA1 or PM2 are activated)	Since VarSome retrieves information from the CGD database, in cases of known inheritance discrepancies, use the following gnomAD cutoffs * to include other variants in the selection: - AR/XL model: <3 homozygotes/hemizygotes in gnomAD exomes&genomes - AD model: <5 heterozygotes in gnomAD exomes&genomes * These rules are used to decide whether variants in genes with AD/AR inheritance should be reported in “Primary” or “Secondary” results in the clinical report (“Primary”: any P/LP variant in genes associated with AD or AR diseases, or VUS in AD genes; “Secondary”: any VUS in AR genes or VUS in AD/AR genes with ≥5 heterozygotes in gnomAD).
BS3 Well-established in vitro or in vivo functional studies show no damaging effect on protein function or splicing	VARIABLE	Consult PUBMED, LOVD and other available databases (Mastermind, LitVar, etc.) to find functional evidence [13].
BS4 Lack of segregation in affected members of a family	NO	Segregation analysis required.
BP1 Missense variant in a gene for which primarily truncating variants are known to cause disease	YES (Mutually exclusive vs. PP2)	Use SUPPORTING as default.
BP2 Observed in trans with a pathogenic variant for a fully penetrant dominant gene/disorder or observed in cis with a pathogenic variant in any inheritance pattern	NO	Segregation analysis required, use SUPPORTING as default. Use STRONG if the condition is confirmed in many individuals (literature or internal evidence) or with different variants.
BP3 In-frame deletions/insertions in a repetitive region without a known function	YES	Use SUPPORTING as default.
BP4 Multiple lines of computational evidence suggest no impact on gene or gene product (conservation, evolutionary, splicing impact, etc.)	YES	- Prediction must be based on at least 3 total and concordant predictors, otherwise exclude the criterion. - Do not assign to any variant with PVS1 activated. - The criterion must not be assigned to canonical splicing variants (±1–2) if PVS1 is assigned. If available, ADA and RF scores ^§^ can be used to assign the criterion to intronic variants. - Do not use ADA and RF scores to assign the criterion to synonymous variants if BP7 is already assigned.
BP5 Variant found in a case with an alternative molecular basis for disease	NO	Segregation analysis or literature evidence required. Use SUPPORTING as default.
BP6 Reputable source recently reports variant as benign, but the evidence is not available to the laboratory to perform an independent evaluation	YES	Exclude the criterion if the variant has “Review status” 0 stars in ClinVar and there are no other submissions in other clinical databases (e.g., LOVD). Be aware that certain UniProt classifications might be outdated.
BP7 A synonymous (silent) variant for which splicing prediction algorithms predict no impact on the splice consensus sequence nor creation of a new splice site AND the nucleotide is not highly conserved	YES	Use SUPPORTING as default.
PATHOGENIC CRITERIA		
ACMG CRITERIA	ASSIGNED BY VARSOME	EXCEPTIONS AND INDICATIONS
PVS1 Null variant (nonsense, frameshift, canonical ±1 or 2 splice sites, initiation codon, single or multiexon deletion) in a gene where LoF is a known mechanism of disease	YES	- Modify the criterion strength according to Abu Tayoun et al. 2018 [10]. - the criterion does not apply to variants in the first/last base of an exon (not considered canonical in the ACMG guidelines). - use SUPPORTING if NMD is not predicted (variant in the last exon or in the last 50 bps of the second-last exon) AND there are no other P/LP variants downstream.
PS1 Same amino acid change as a previously established pathogenic variant regardless of nucleotide change	YES	Use STRONG as default, reduce to SUPPORTING if the alternative variant is classified as likely pathogenic. Always check interpretation of alternative variant.
PS2: De novo (confirmed maternity and paternity) in a patient with the disease and no family history	NO	Segregation analysis required.
PS3: Well-established in vitro or in vivo functional studies supporting a damaging effect on the gene or gene product	VARIABLE	- Consult PUBMED, LOVD, and other available databases (Mastermind, LitVar, etc.) to identify functional evidence - Modify criterion strength according to evidence relevance [13]
PS4 The prevalence of the variant in affected individuals is significantly higher than in controls	NO	- Use the criterion at STRONG level if prevalence data (cases/controls) are available - Use the criterion when the variant has been reported in at least 5 unrelated affected individuals in the laboratory
PM1: Located in a mutational hotspot and/or critical and well-established functional domain (e.g., the active site of an enzyme) without benign variation	YES	- Use MODERATE as default. - Reduce to SUPPORTING with <10 variants in the domain
PM2: Absent in controls (or extremely low frequency if recessive) in Exome Sequencing Project, 1000 Genomes Project, or Exome Aggregation Consortium	YES	Use MODERATE as default Since VarSome employs the CGD database, in case of known inheritance discrepancies, use the following gnomAD cutoffs * to include other variants in the selection: - AR/XL model: <3 homozygotes/hemizygotes in gnomAD exomes&genomes - AD model: <5 heterozygotes in gnomAD exomes&genomes * These rules are used to decide if variants in genes with AD/AR inheritance should be reported in “Primary” or “Secondary” results (“Primary”: any P/LP variant in genes associated with AD or AR disease, or VUS in AD genes; “Secondary”: any VUS in AR genes, or VUS in AD/AR genes with ≥5 heterozygotes in gnomAD).
PM3 For recessive disorders, detected in trans with a pathogenic variant	NO	- Use MODERATE as default - Use SUPPORTING if variant found in trans with only one other LP/P variant in one affected individual or for homozygous genotypes [29] - Upgrade to STRONG if found in trans with multiple different pathogenic variants or in multiple affected individuals (in the literature or in using internal segregation evidence).
PM4 Protein length changes as a result of in-frame deletions/insertions in a non-repeat region or stop-loss variants	YES (not applicable if PVS1 is enabled)	Use MODERATE as default.
PM5: Novel missense change at an amino acid residue where a different missense change determined to be pathogenic has been seen before.	YES	Reduce to SUPPORTING in dubious cases, be aware that certain UniProt classification might be outdated, therefore always check interpretation of different missense changes.
PM6: Assumed de novo, but without confirmation of paternity and maternity.	NO	It is possible to modify criterion strength according to the compatibility of the proband’s phenotype with the disease associated with the gene and if the variant has been found de novo in other non-consanguineous individuals in the internal database. (Further implementations ongoing to refine grading).
PP1: Cosegregation with disease in multiple affected family members in a gene definitively known to cause the disease.	NO	Use: - STRONG: ≥4 total affected persons including the one tested - MODERATE: 2–3 affected persons including the tested individual - SUPPORTING: 1 affected person including the tested individual
PP2: Missense variant in a gene that has a low rate of benign missense variation and in which missense variants are a common mechanism of disease.	YES	Use SUPPORTING as default.
PP3: Multiple lines of computational evidence support a deleterious effect on the gene or gene product (conservation, evolutionary, splicing impact, etc.)	YES	- Prediction must be based on at least 3 total and concordant predictors, otherwise exclude the criterion. - Do not assign to any variant with PVS1 activated. - The criterion must not be assigned to canonical splicing variants (±1–2) if PVS1 is assigned. If available, ADA and RF scores^§^ can be used to assign the criterion to intronic variants.
PP4: Patient’s phenotype or family history is highly specific for a disease with a single genetic etiology.	NO	- Use SUPPORTING for diseases with no more than 5 associated genes (e.g., Stargardt disease) - Use MODERATE for true single-gene disorders (e.g., CHM for choroideremia).
PP5: Reputable source recently reports variant as pathogenic, but the evidence is not available to the laboratory to perform an independent evaluation.	YES	Exclude the criterion if the variant has “Review status” 0 stars in ClinVar and there are no other submissions in other clinical databases (e.g., LOVD). Be aware that certain UniProt classifications might be outdated.

^§^ Cutoffs: ADA score > 0.708; RF score > 0.515.

## Data Availability

The data presented in this study are available on request from the corresponding author.

## References

[1] Kazazian H., Boehm C., Seltzer W. (2000). ACMG recommendations for standards for interpretation of sequence variations. Genet. Med..

[2] Richards C.S., Bale S., Bellissimo D.B., Das S., Grody W.W., Hegde M.R., Lyon E., Ward B.E. (2008). ACMG recommendations for standards for interpretation and reporting of sequence variations: Revisions 2007. Genet. Med..

[3] Richards S., Aziz N., Bale S., Bick D., Das S., Gastier-Foster J., Grody W., Hegde M., Lyon E., Spector E. (2015). Standards and guidelines for the interpretation of sequence variants: A joint consensus recommendation of the American College of Medical Genetics and Genomics and the Association for Molecular Pathology. Genet. Med..

[4] Amendola L.M., Jarvik G.P., Leo M.C., McLaughlin H.M., Akkari Y., Amaral M.D., Berg J.S., Biswas S., Bowling K.M., Conlin L.K. (2016). Performance of ACMG-AMP Variant-Interpretation Guidelines among Nine Laboratories in the Clinical Sequencing Exploratory Research Consortium. Am. J. Hum. Genet..

[5] Pepin M.G., Murray M.L., Bailey S., Leistritz-Kessler D., Schwarze U., Byers P.H. (2016). The challenge of comprehensive and consistent sequence variant interpretation between clinical laboratories. Genet. Med..

[6] Nykamp K., Anderson M., Powers M., Garcia J., Herrera B., Ho Y.Y., Kobayashi Y., Patil N., Thusberg J., Westbrook M. (2017). Sherloc: A comprehensive refinement of the ACMG-AMP variant classification criteria. Genet. Med..

[7] Karbassi I., Maston G.A., Love A., Divincenzo C., Braastad C.D., Elzinga C.D., Bright A.R., Previte D., Zhang K., Rowland C.M. (2016). A standardized DNA variant scoring system for pathogenicity assessments in Mendelian disorders. Hum. Mutat..

[8] Jarvik G.P., Browning B.L. (2016). Consideration of cosegregation in the pathogenicity classification of genomic variants. Am. J. Hum. Genet..

[9] Tavtigian S.V., Greenblatt M.S., Harrison S.M., Nussbaum R.L., Prabhu S.A., Boucher K.M., Biesecker L.G. (2018). Modeling the ACMG/AMP variant classification guidelines as a Bayesian classification framework. Genet. Med..

[10] Abou Tayoun A.N., Pesaran T., DiStefano M.T., Oza A., Rehm H.L., Biesecker L.G., Harrison S.M. (2018). Recommendations for interpreting the loss of function PVS1 ACMG/AMP variant criterion. Hum. Mutat..

[11] Ghosh R., Harrison S.M., Rehm H.L., Plon S.E., Biesecker L.G. (2018). Updated recommendation for the benign stand-alone ACMG/AMP criterion. Hum. Mutat..

[12] Biesecker L.G., Harrison S.M. (2018). The ACMG/AMP reputable source criteria for the interpretation of sequence variants. Genet. Med..

[13] Brnich S.E., Tayoun A.N.A., Couch F.J., Cutting G.R., Greenblatt M.S., Heinen C.D., Kanavy D.M., Luo X., McNulty S.M., Starita L.M. (2019). Recommendations for application of the functional evidence PS3/BS3 criterion using the ACMG/AMP sequence variant interpretation framework. bioRxiv.

[14] Oza A.M., DiStefano M.T., Hemphill S.E., Cushman B.J., Grant A.R., Siegert R.K., Shen J., Chapin A., Boczek N.J., Schimmenti L.A. (2018). Expert specification of the ACMG/AMP variant interpretation guidelines for genetic hearing loss. Hum. Mutat..

[15] Gelb B.D., Cavé H., Dillon M.W., Gripp K.W., Lee J.A., Mason-Suares H., Rauen K.A., Williams B., Zenker M., Vincent L.M. (2018). ClinGen’s RASopathy Expert Panel consensus methods for variant interpretation. Genet. Med..

[16] Lee K., Krempely K., Roberts M.E., Anderson M.J., Carneiro F., Chao E., Dixon K., Figueiredo J., Ghosh R., Huntsman D. (2018). Specifications of the ACMG/AMP variant curation guidelines for the analysis of germline CDH1 sequence variants. Hum. Mutat..

[17] Johnston J.J., Dirksen R.T., Girard T., Gonsalves S.G., Hopkins P.M., Riazi S., Saddic L.A., Sambuughin N., Saxena R., Stowell K. (2021). Variant curation expert panel recommendations for RYR1 pathogenicity classifications in malignant hyperthermia susceptibility. Genet. Med..

[18] McCormick E.M., Lott M.T., Dulik M.C., Shen L., Attimonelli M., Vitale O., Karaa A., Bai R., Pineda-Alvarez D.E., Singh L.N. (2020). Specifications of the ACMG/AMP standards and guidelines for mitochondrial DNA variant interpretation. Hum. Mutat..

[19] Shen J., Oza A.M., del Castillo I., Duzkale H., Matsunaga T., Pandya A., Kang H.P., Mar-Heyming R., Guha S., Moyer K. (2019). Consensus interpretation of the p.Met34Thr and p.Val37Ile variants in GJB2 by the ClinGen Hearing Loss Expert Panel. Genet. Med..

[20] Harrison S.M., Dolinsky J.S., Knight Johnson A.E., Pesaran T., Azzariti D.R., Bale S., Chao E.C., Das S., Vincent L., Rehm H.L. (2017). Clinical laboratories collaborate to resolve differences in variant interpretations submitted to ClinVar. Genet. Med..

[21] Harrison S.M., Dolinksy J.S., Chen W., Collins C.D., Das S., Deignan J.L., Garber K.B., Garcia J., Jarinova O., Knight Johnson A.E. (2018). Scaling resolution of variant classification differences in ClinVar between 41 clinical laboratories through an outlier approach. Hum. Mutat..

[22] Kopanos C., Tsiolkas V., Kouris A., Chapple C.E., Albarca Aguilera M., Meyer R., Massouras A. (2019). VarSome: The human genomic variant search engine. Bioinformatics.

[23] Sorrentino E., Cristofoli F., Modena C., Marceddu G. (2021). Integrate VarSome API for automate ACMG clinical variant interpretation. Acta Biomed..

[24] Marceddu G., Dallavilla T., Guerri G., Manara E., Chiurazzi P., Bertelli M. (2019). PipeMagi: An integrated and validated workflow for analysis of NGS data for clinical diagnostics. Eur. Rev. Med. Pharmacol. Sci..

[25] Marceddu G., Dallavilla T., Xhuvani A., Daja M., De Antoni L., Casadei A., Bertelli M. (2020). AppMAGI: A complete laboratory information management system for clinical diagnostics. Acta Biomed..

[26] Liu X., Li C., Mou C., Dong Y., Tu Y. (2020). dbNSFP v4: A comprehensive database of transcript-specific functional predictions and annotations for human nonsynonymous and splice-site SNVs. Genome Med..

[27] McLaren W., Gil L., Hunt S.E., Riat H.S., Ritchie G.R.S., Thormann A., Flicek P., Cunningham F. (2016). The Ensembl Variant Effect Predictor. Genome Biol..

[28] Karczewski K.J., Francioli L.C., Tiao G., Cummings B.B., Alföldi J., Wang Q., Collins R.L., Laricchia K.M., Ganna A., Birnbaum D.P. (2020). The mutational constraint spectrum quantified from variation in 141,456 humans. Nature.

[29] Ellard S., Baple E.L., Berry I., Forrester N., Turnbull C., Owens M., Eccles D.M., Abbs S., Scott R., Deans Z.C. (2019). ACGS Best Practice Guidelines for Variant Classification 2019. https://www.acgs.uk.com/news/acgs-best-practice-guidelines-for-variant-classification-2019/.

[30] Cremers F.P.M., Lee W., Collin R.W.J., Allikmets R. (2020). Clinical spectrum, genetic complexity and therapeutic approaches forretinal disease caused by ABCA4 mutations. Prog. Retin. Eye Res..

[31] Zernant J., Lee W., Collison F.T., Fishman G.A., Sergeev Y.V., Schuerch K., Sparrow J.R., Tsang S.H., Allikmets R. (2017). Frequent hypomorphic alleles account for a significant fraction of ABCA4 disease and distinguish it from age-related macular degeneration. J. Med. Genet..

[32] Fukai K., Holmes S.A., Lucchese N.J., Siu V.M., Weleber R.G., Schnur R.E., Spritz R.A. (1995). Autosomal recessive ocular albinism associated with a functionally significant tyrosinase gene polymorphism. Nat. Genet..

[33] Simeonov D.R., Wang X., Wang C., Sergeev Y., Dolinska M., Bower M., Fischer R., Winer D., Dubrovsky G., Balog J.Z. (2013). DNA Variations in oculocutaneous albinism: An updated mutation list and current outstanding issues in molecular diagnostics. Hum. Mutat..

[34] Xiang J., Peng J., Baxter S., Peng Z. (2020). AutoPVS1: An automatic classification tool for PVS1 interpretation of null variants. Hum. Mutat..

[35] Ellard S., Baple E.L., Callaway A., Berry I., Forrester N., Turnbull C., Owens M., Eccles D.M., Abbs S., Scott R. (2020). ACGS Best Practice Guidelines for Variant Classification in Rare Disease 2020. https://www.acgs.uk.com/media/11631/uk-practice-guidelines-for-variant-classification-v4-01-2020.pdf.

[36] Houge G., Laner A., Cirak S., de Leeuw N., Scheffer H., den Dunnen J.T. (2021). Stepwise ABC system for classification of any type of genetic variant. Eur. J. Hum. Genet..

[37] MacDonald I.M., Lee T., Lawrence J., Adam M.P., Ardinger H.H., Pagon R.A., Wallace S.E., Bean L.J.H., Stephens K., Amemiya A. (2020). Bestrophinopathies.

[38] Majewski I.J., Kluijt I., Cats A., Scerri T.S., De Jong D., Kluin R.J.C., Hansford S., Hogervorst F.B.L., Bosma A.J., Hofland I. (2013). An α-E-catenin (CTNNA1) mutation in hereditary diffuse gastric cancer. J. Pathol..

[39] Clark D.F., Michalski S.T., Tondon R., Nehoray B., Ebrahimzadeh J., Hughes S.K., Soper E.R., Domchek S.M., Rustgi A.K., Pineda-Alvarez D. (2020). Loss-of-function variants in CTNNA1 detected on multigene panel testing in individuals with gastric or breast cancer. Genet. Med..

[40] Saksens N.T.M., Krebs M.P., Schoenmaker-Koller F.E., Hicks W., Yu M., Shi L., Rowe L., Collin G.B., Charette J.R., Letteboer S.J. (2016). Mutations in CTNNA1 cause butterfly-shaped pigment dystrophy and perturbed retinal pigment epithelium integrity. Nat. Genet..

[41] Tanner A., Chan H.W., Pulido J.S., Arno G., Ba-Abbad R., Jurkute N., Robson A.G., Egan C.A., Knight H., Calcagni A. (2021). Clinical and genetic findings in CTNNA1-associated macular pattern dystrophy. Ophthalmology.

